# Selenomethionine Inhibited HADV-Induced Apoptosis Mediated by ROS through the JAK-STAT3 Signaling Pathway

**DOI:** 10.3390/nu16121966

**Published:** 2024-06-20

**Authors:** Chuqing Li, Xia Liu, Jiali Li, Jia Lai, Jingyao Su, Bing Zhu, Buyun Gao, Yinghua Li, Mingqi Zhao

**Affiliations:** 1 Center Laboratory, Guangzhou Women and Children’s Medical Center, Guangzhou Medical University, Guangzhou 510120, China; lcq@stu.gzhmu.edu.cn (C.L.); liuxia@stu.gzhmu.edu.cn (X.L.); 2023210292@stu.gzhmu.edu.cn (J.L.); 2022210293@stu.gzhmu.edu.cn (J.L.); sujingyao@stu.gzhmu.edu.cn (J.S.); zhubing@gzhmu.edu.cn (B.Z.); 2 School of Pharmacy, Fudan University, Shanghai 200437, China; 19301030054@fudan.edu.cn

**Keywords:** selenomethionine, adenovirus, ROS, apoptosis

## Abstract

Adenovirus (HAdV) can cause severe respiratory infections in children and immunocompromised patients. There is a lack of specific therapeutic drugs for HAdV infection, and the study of anti-adenoviral drugs has far-reaching clinical implications. Elemental selenium can play a specific role as an antioxidant in the human immune cycle by non-specifically binding to the amino acid methionine in body proteins. Methods: The antiviral mechanism of selenomethionine was explored by measuring cell membrane status, intracellular DNA status, cytokine secretion, mitochondrial membrane potential, and ROS production. Conclusions: Selenomethionine improved the regulation of ROS-mediated apoptosis by modulating the expression of Jak1/2, STAT3, and BCL-XL, which led to the inhibition of apoptosis. It is anticipated that selenomethionine will offer a new anti-adenoviral therapeutic alternative.

## 1. Introduction

With a diameter of 70–100 nm, HAdV is a double-stranded, envelope-less DNA virus [1]. After being mainly isolated from recruits suffering from acute febrile respiratory infections, HAdVs were subsequently associated with many clinical manifestations [2]. HAdV infections are more common in young children due to the lack of humoral immunity, and the infection spreads easily. In some cases, it is highly contagious. Although the clinical course of the disease is usually mild and self-limited, local outbreaks of HAdV, epidemics of infection, and a severe course of the disease can occur in healthy children or adults in closed or crowded environments, especially in military recruits. Sometimes, transmission is even more likely in severely immunocompromised individuals (such as organ transplant recipients and people infected with the human immunodeficiency virus), resulting in fatal results [1,2,3]. HAdV can cause different clinical manifestations, ranging from latent infection or benign upper respiratory tract disease to necrotizing bronchiolitis and even diffuse fatal disease in immunocompromised patients. In addition to respiratory tract involvement, it also involves keratoconjunctivitis, urinary tract infection, encephalitis, myocarditis, and so on [1]. The activation of the immune system and the production of a large number of chemokines and cytokines play a role in the activation of inflammation and thus may play a major role in the pathogenesis of tissue damage [4]. It has been demonstrated that both chemotactic and non-chemotactic cytokines are crucial for host defense. These cytokines include tumor necrosis factor-alpha (TNFα), IL-1, IL-6, IL-8, IL-10, IL-12, interferon-gamma (IFNγ), and granulocyte colony-stimulating factor [3]. After HAdV-1 and -2, HAdV-7 is the third most prevalent serotype that has been reported to the World Health Organization. It is still one of the main serotypes that have been found to be associated with disease worldwide [3]. HAdV-7 infection presents with acute respiratory disease, pharyngoconjunctival fever, bronchitis, necrotizing bronchiolitis, or pneumonia. In comparison to other serotypes, HAdV-7 seems to be more virulent. Both adults and children who are immunocompetent can get fatal pneumonia. The two main serotypes of HAdV linked to pediatric acute respiratory illnesses in Asia are -7 and -3. Types 1 through 7 of the HAdV virus are most frequently linked to acute respiratory illnesses in children. The serotypes HAdV-1 through 7, -21, and -14 are the most frequently linked to acute respiratory diseases in adults [3].

HAdV can be detected at the site of infection (e.g., nasopharyngeal inhalation, swabs, washes, bronchoalveolar lavage, urine, feces, blood) by direct or indirect immunofluorescence, routine or shell vial culture, or PCR. Viral cultures by traditional techniques are the gold standard but may not be sensitive to certain samples (e.g., blood) and may take up to 21 days to produce cytopathic effects [5,6]. The PCR detection of HAdV DNA in plasma, urine, or other clinical specimens is currently the most commonly used diagnostic method and is very sensitive for disseminated disease. And the real-time PCR quantification of the viral load is a valid indicator for assessing treatment response [7,8].

In many cases, supportive care and, where appropriate, reduced immunosuppression are the primary management strategies for patients with HAdV infection. There are currently no specific antiviral drugs approved for the treatment of HAdV infection [9]. Instead, the treatment of severe HAdV infection revolves around the off-label use of broad-spectrum antivirals such as cidofovir, ganciclovir, and ribavirin. However, most evidence suggests that ribavirin and ganciclovir are of little value as HAdV treatment agents. Currently, cidofovir is used as a standard treatment. Originally, cidofovir was developed for the treatment of cytomegalovirus infections, but there is a large body of evidence to support its use as a treatment for adenovirus [3,10,11,12].

Selenium exists in two forms in an organism; one is inorganic selenium and the other is organic selenium [13]. Organic and inorganic selenium can be directly absorbed by the human body. However, organic selenium has a higher absorption, conversion, and utilization rate than inorganic selenium and can be stored in human tissues [14,15]. In addition to nanoselenium, selenium is deposited in tissues in the form of selenoproteins. Inorganic selenium is mostly consumed directly through metabolism and has a greater toxic effect on the body than organic selenium. Organic selenium is categorized into two groups, seleno-substituted amino acids (including selenocystine and selenomethionine) and selenoproteins (including the selenium-containing protein glutathione peroxidase and others) [16]. By non-specific binding to the amino acid methionine in the body protein, selenium can play a specific role in the human immune cycle as an antioxidant, such as antioxidant, anti-inflammatory, anti-mutagenesis, anti-cancer, antiviral, anti-bacterial roles, etc. [17,18,19,20]. Selenomethionine, as the main chemical form of dietary selenium in our daily supplement, has been widely used in various health foods [21]. The U.S. Food and Nutrition Board recommends that adults consume 50–200 MCG/day of selenium, and the World Health Organization recommends that the minimum dietary selenium requirement be 40 MCG/day and nutritional supplements 50–250 MCG/day [21]. Most of the selenoproteins identified to the present day are enzymes with important functions, such as glutathione peroxidase (GPx), iodothyronine deiodinase (ID), and thioredoxin reductase (TrxR). Selenoproteins generally contain selenocysteine, and selenium in the form of Sec constitutes the active center of selenoenzymes [16]. The antioxidant effect of selenium is mainly mediated by glutathione peroxidase. It often forms a system for inhibiting lipid peroxidation together with other antioxidant enzymes, scavenging free radicals generated by oxidative processes and preventing lipid oxidation.

In multicellular organisms, apoptosis is a crucial self-stabilizing mechanism that eliminates undesirable or aberrant cells. It also plays a significant role in normal physiological processes like embryonic development, internal environment homeostasis, and pathogen resistance. Disorders of this mechanism can induce various diseases, such as cancer, autoimmune diseases, cardiovascular diseases, and neurodegenerative diseases [22]. There is a close relationship between oxidative stress and apoptosis. Oxidative stress can induce apoptosis through multiple pathways. First, oxidative stress may result in the lipid peroxidation of cell membranes, which may modify the composition and functionality of cell membranes and ultimately result in apoptosis. Second, oxidative stress can harm intracellular proteins through oxidative stress, which can change the structure and function of proteins and ultimately result in apoptosis [22,23,24]. Additionally, oxidative stress can damage intracellular DNA oxidatively, changing its structure and function and ultimately resulting in apoptosis. Intracellular ROS are mainly produced by mitochondria, and normally, mitochondria and the rest of cellular components are equipped with functional antioxidant systems to ensure that ROS are maintained at low levels [23]. ROS are involved as signaling molecules in a wide range of physiological activities such as cell proliferation, differentiation apoptosis, and so on. And the state of oxidative stress is actually a state of high ROS levels. ROS exceeds the scavenging capacity of the antioxidant system, resulting in abnormal physiological activities of mitochondria and even cells. In our study, adenovirus induces apoptosis after infecting cells [25,26]. The conversion of selenomethionine to selenoprotein may act as an antioxidant through free radical scavenging activity. It can generate an antioxidant effect thereby reducing oxidative damage in different internal organs [21].

The purpose of this article is to study the signaling pathway of selenomethionine inhibiting apoptosis induced by adenovirus type 7.

## 2. Results and Discussion

### 2.1. Detection of Optimal Low-Toxicity Antiviral Concentrations of Selenomethionine

**Figure 1:**
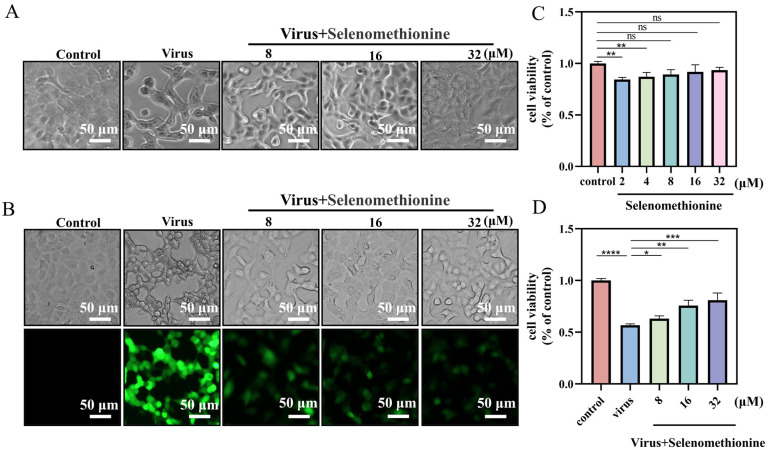
Selenomethionine’s optimal low-toxicity antiviral concentration in A549 cells. (**A**) Changes in the status of HAdV-7-infected A549 cells show the antiviral capacity of selenomethionine in the concentration range of 8–32 μM. (**B**) Fluorescence changes in rAD7EGFP-infected A549 cells showing the antiviral ability of selenomethionine in the concentration range of 8–32 μM. (**C**) The CCK-8 assay detects the viability of selenomethionine in the concentration range of 2–32 μM on A549 cells. (**D**) The viability of HAdV-7-infected A549 cells as detected by the CCK-8 assay increased with increasing concentrations of selenomethionine in the 8–32 μM concentration range. Error lines indicate the mean ± standard deviation (n = 3), and confidence intervals are at a 95% level. Differences between data were statistically significant when *p* < 0.05 (*), *p* < 0.01 (**), *p* < 0.001 (***), or *p* < 0.0001 (****).

This study aimed to explore the antiviral effect of selenomethionine. The CCK-8 assay was used to assess the toxicity of selenomethionine to both uninfected A549 cells and those infected with HAdV-7. Selenomethionine showed no significant cytotoxic effects on A549 cells in the concentration range of 8–32 μM compared to the control (Figure 1C). Therefore, selenomethionine in this concentration range was selected for subsequent experiments to understand its antiviral efficiency. The fluorescent virus rAD7EGFP, which produces enhanced green fluorescence when propagated in vivo, was employed in this experiment to examine the antiviral effect of selenomethionine. The fluorescence changes in rAD7EGFP-infected A549 cells after selenomethionine treatment were observed under a fluorescence microscope in the concentration range of 8–32 μM. After the HAdV-7 infection of A549 cells, most of the cells excited green fluorescence, and the higher the fluorescence intensity, the higher the viral load. As the concentration of selenomethionine increased, the number of cells with green fluorescence decreased, the fluorescence intensity weakened, and the viral load decreased (Figure 1B). The antiviral efficiency of selenomethionine was quantified by fully automated zymography. The viability of HAdV-7-infected A549 cells as detected by the CCK-8 assay increased with increasing concentrations of selenomethionine in the 8–32 μM concentration range (Figure 1D). The experimental findings demonstrated that the cell survival rate of the virus group was 60.1% compared with the control group. The cell survival rate of the virus + selenomethionine group was 63.17%, 75.78%, and 80.93%, respectively. The survival rate of HAdV-7-infected cells rose as the increasing concentration of selenomethionine. Selenomethionine increased the antiviral efficiency in a dose-dependent manner. 

### 2.2. Detection of Selenomethionine Regulates Cell Membrane Phosphatidylserine Translocation and Cell Membrane Permeability

**Figure 2:**
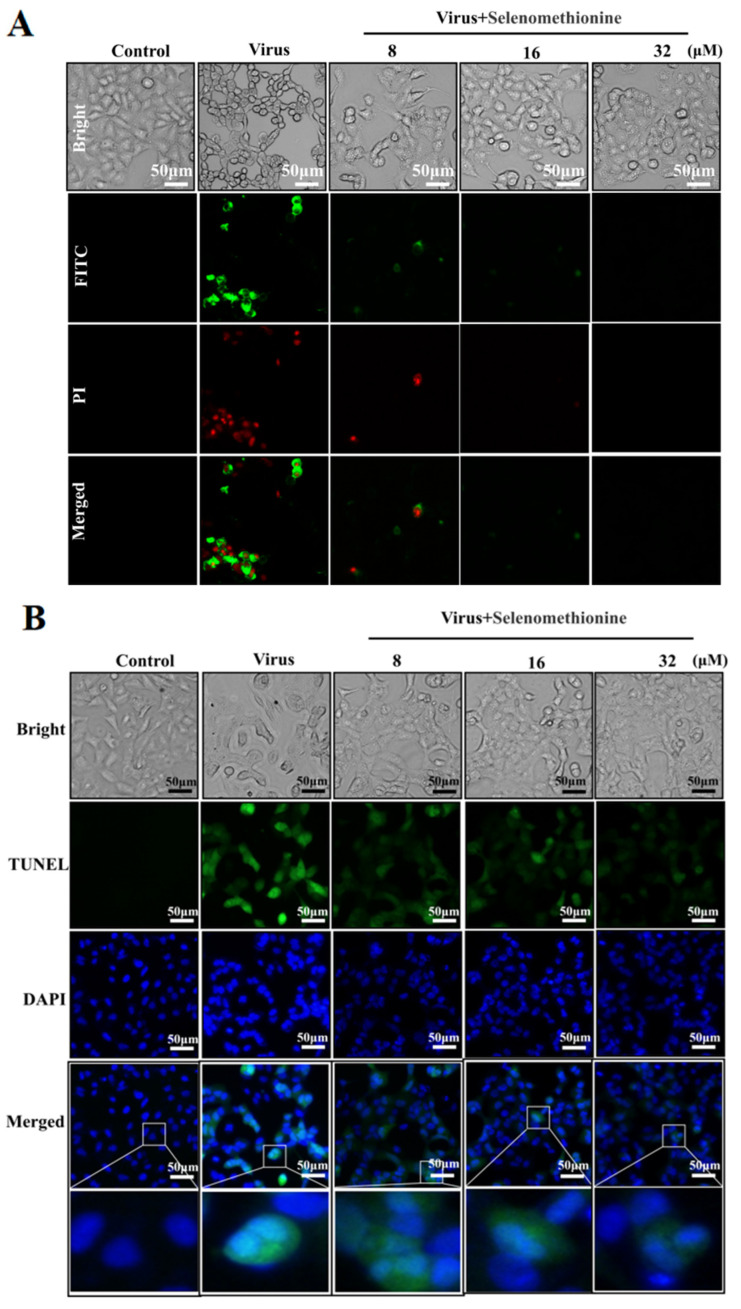
Selenomethionine’s inhibitory action on DNA damage and apoptosis brought on by HAdV-7 infection. (**A**) Results of cells stained with Annexin V-FITC and PI from several experimental groups. Phosphatidylserine translocation and A549 cell membrane’s permeability were found using fluorescence microscopy. (**B**) Results of cells in different experimental groups stained with TUNEL-DAPI double staining. DNA damage and nucleus deformation in A549 cells were detected by fluorescence microscopy. Concentrations of SeC were 8 μM, 16 μM, and 32 μM, respectively.

Phosphatidylserine is predominantly found on the inside of cell membranes. In the early stages of apoptosis, different types of cells ectopically transfer phosphatidylserine to the outside of the cell membrane. The selective binding of Annexin V to phosphatidylserine makes it possible to detect the ectoposition of phosphatidylserine, an important feature of apoptosis, very simply and directly by fluorescence microscopy. Propidium iodide stains necrotic cells or cells with the loss of cell membrane integrity in the late stages of apoptosis with red fluorescence. For necrotic cells, because cell membrane integrity has been lost, Annexin V-FITC can enter the cytoplasm and bind to phosphatidylserine located on the inner side of the cell membrane, thus also causing necrotic cells to show green fluorescence. Early apoptotic cells showed positive Amexin V-FITC staining and negative PI staining. Late apoptotic and necrotic cells showed double-positive Amexin VFITC staining and PI staining [27,28]. Low-fluorescence intensity red and green fluorescence was seen in normal controls. The group infected with the adenovirus exhibited intense red and green fluorescent signals, signifying that the cells were in apoptosis. Red and green fluorescence intensities were reduced in HAdV-7-infected A549 cells treated with selenomethionine in comparison to the virus group (Figure 2). According to the experimental findings, HAdV-7 infection caused cells’ regular cell cycle to be disrupted, allowing the cells to enter an aberrant apoptotic and necrotic cycle. The selenomethionine have successfully reduced necrosis and apoptosis brought on by HAdV-7 infection. 

### 2.3. Detection of Inhibition of DNA Fragmentation Damage by Selenomethionine

A549 cells infected with HAdV-7 showed large amounts of DNA breakage damage localized in green fluorescence and occurring predominantly in the nucleus, with significant nuclear deformation localized in blue fluorescence. When selenomethionine treated HAdV-7-infected A549 cells, the cellular green fluorescence in the concentration range of 8–32 μM was significantly reduced with the increase in selenomethionine concentration in comparison with the viral group (Figure 2B). Research has shown that selenomethionine reduced the damage to DNA fragmentation caused by HAdV-7 infection in A549 cells.

### 2.4. Detection of Selenomethionine Altered Mitochondrial Membrane Potential (∆Ψm)

**Figure 3:**
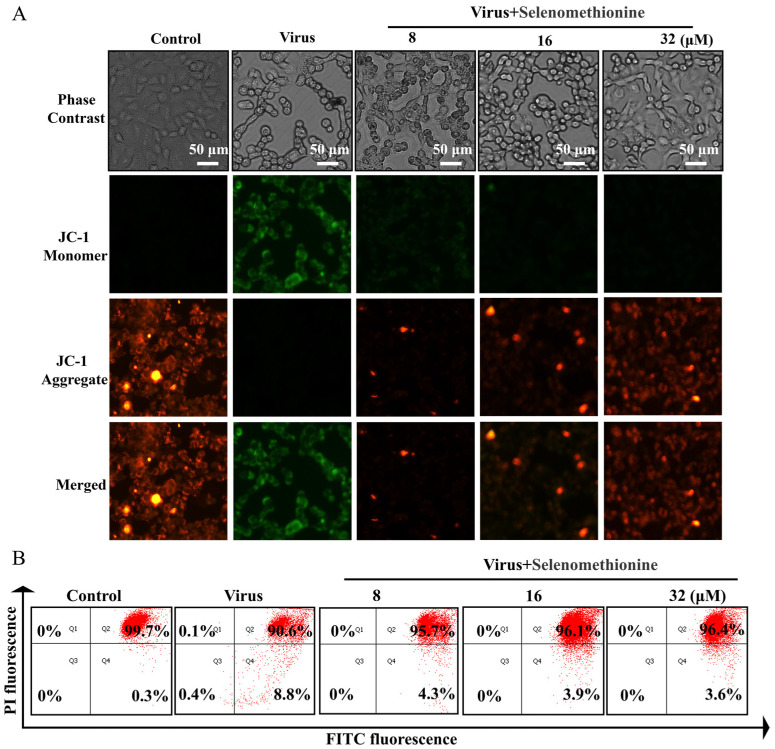
Selenomethionine repairs mitochondrial dysfunction induced by HAdV-7 infection. (**A**) The JC-1 Mitochondrial Membrane Potential Assay Kit was utilized to identify alterations in mitochondrial membrane potential, while a fluorescence microscope was employed to observe changes in fluorescence. (**B**) The quantification of cells with different fluorescence intensities by flow cytometry. The concentration of selenomethionine were 8 μM, 16 μM, and 32 μM, in that order.

The JC-1 fluorescent probe provides the rapid and sensitive detection of changes in cellular mitochondrial membrane potential. Changes in mitochondrial membrane potential can reflect the functional status of mitochondria to a certain extent. In the early stages of apoptosis, a landmark event is the decrease in mitochondrial membrane potential [29]. The shift in JC-1’s fluorescence from red to green indicates a drop in mitochondrial membrane potential and can serve as a marker for the early stages of apoptosis [30]. Green fluorescence with low fluorescence intensity and red fluorescence with high fluorescence intensity were seen in the normal controls, according to the experimental data. The adenovirus group’s mitochondria displayed red fluorescence with low fluorescence intensity and green fluorescence with high fluorescence intensity, suggesting that mitochondrial function was compromised, and the cells were close to apoptosis. When selenomethionine was administered to A549 cells infected with HAdV-7, the intensity of cellular mitochondria’s red fluorescence increased, and their green fluorescence decreased (Figure 3A). The percentage of cells in each experimental group at different phases was determined using flow cytometry. In the control group, 99.7% of normal cells and 0.3% of apoptotic cells were characterized by red fluorescence intensity, while in the viral group, 90.6% of normal cells and 8.8% of apoptotic cells were characterized by green fluorescence intensity. The virus group showed a substantial rise of 8.5% in the green fluorescence intensity of apoptotic cells and a significant decrease of 9.1% in the red fluorescence intensity of normal cells in comparison to the control group. However, the green fluorescence intensity of apoptotic cells of A549 cells infected with HAdV-7 after treatment with selenomethionine in the concentration ranges of 8 μM, 16 μM, and 32 μM significantly decreased to 4.3%, 3.9%, and 3.6%, respectively. The intracellular fluorescence changes observed by fluorescence microscopy were consistent with the results of flow cytometry (Figure 3B).

### 2.5. Detection of Selenomethionine Inhibits ROS Generation

**Figure 4:**
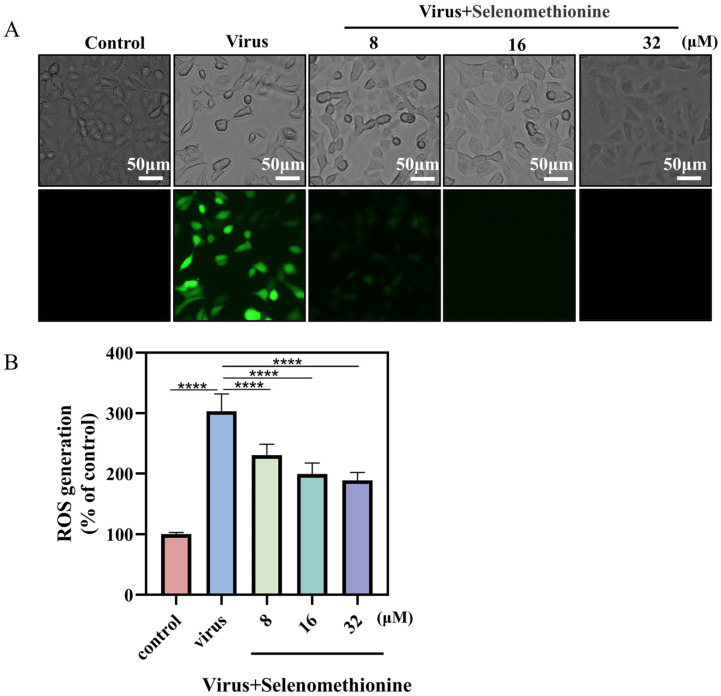
Selenomethionine reduces HAdV-7 infection-induced ROS overproduction. (**A**) An optical microscope was used to observe the cell morphology of different experimental groups and fluorescence microscope to detect the production of ROS. (**B**) Using a fully automated enzyme labeler, the absorbance was determined at a 488 nm excitation wavelength and 525 nm emission wavelength. The level of ROS was shown by the fluorescence intensity. The concentration of selenomethionine were 8 μM, 16 μM, and 32 μM, in that order. *p* < 0.0001 (****) indicates that the difference is statistically significant.

The theory behind the reactive oxygen species (ROS) assay is that non-fluorescent DCFH can be oxidized by intracellular reactive oxygen species to form fluorescent DCF and that the level of ROS is directly proportional to the intensity of green fluorescence [31]. When comparing the viral + selenomethionine group to the virus group, there was a notable decrease in the intensity of green fluorescence (Figure 4A). Semi-quantitative fluorescence readings from fully automated enzyme markers showed a significant increase in ROS production to 303% in the virus group compared to the control group, whereas in the virus + selenomethionine group, the ROS production in the cells decreased to 189% as the concentration of selenomethionine was increased (Figure 4B). The experimental results showed that intracellular ROS generation was decreased following selenomethionine administration, as were ROS-mediated DNA damage and apoptosis. The aforementioned experimental findings led us to the conclusion that excessive intracellular ROS could damage DNA and trigger a number of apoptotic signaling pathways.

### 2.6. Detection of Cytokines in Cell Culture Supernatants

Pro-inflammatory cytokines are secreted by Th1 cells, CD4+ T cells, macrophages, and dendritic cells, and the key pro-inflammatory cytokines are IL-1, IL-6, and TNF-α. They tightly regulate cell-mediated immune responses and play an important role in modulating the immune system. Pro-inflammatory cytokines typically regulate the growth, activation, differentiation, and homing of immune cells to sites of infection, ultimately controlling and eradicating intracellular pathogens, including viruses [32]. TNF-α plays an important role in local and circulating inflammatory responses. TNF-α triggers the expression of vascular endothelial cells, enhances the expression of adhesion molecules by leukocytes, and promotes the infiltration of immune cells. TNF-α is able to increase the infiltration of lymphocytes to the site of infection, which plays a crucial role in the early response to antiviral infections [33,34]. The importance of IFN-γ in the immune system derives in part from its ability to directly inhibit viral replication and, most importantly, from its immunostimulatory and immunomodulatory effects, which induce the expression of viral antigens in virus-infected cells and increase the immune system’s role in recognizing and killing infected cells, thereby resisting viral infection. In addition, IL-8 also enhances IFN-γ secretion to attract monocytes and T lymphocytes to induce apoptosis and clear infected cells.

**Figure 5:**
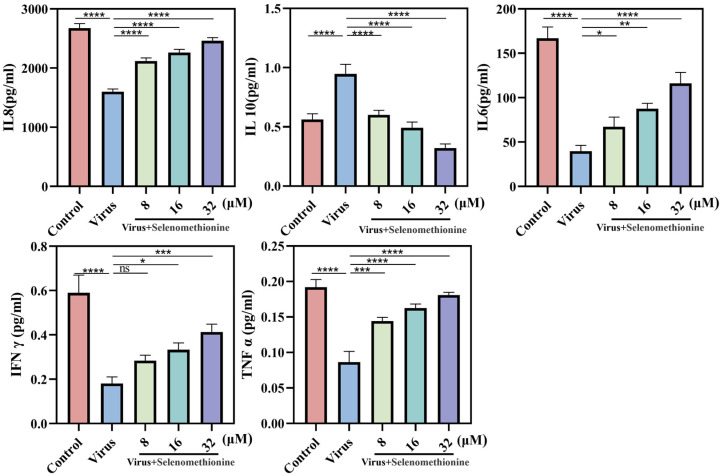
The level of cytokines released by A549 cells infected with HAdV-7 is regulated by selenomethionine. The levels of IL-8, IL-10, IL-6, TNF-α, and IFN-γ that cells in various experimental groups secreted. The concentrations of selenomethionine were 8 μM, 16 μM, and 32 μM, respectively. *p* < 0.05 (*), *p* < 0.01 (**), *p* < 0.001 (***), and *p* < 0.0001 (****) indicated that the differences were statistically significant.

In our experiment, the cytokines of the A549 cell culture supernatant after HAdV-7 infection and selenomethionine treatment were detected. Following treatment with varying medication dosages, IL-8, IL-6, TNF-α, and IFN-γ were increased, and IL-10 was significantly decreased in comparison to the viral group (Figure 5).

We hypothesized that selenomethionine promoted the secretion of cytokines that may be involved in antiviral effects (e.g., IL-8, IL-6, TNF-α, and IFN-γ) and inhibited the secretion of cytokines that may be involved in immunosuppression, such as IL-10.

### 2.7. Inhibition of Apoptosis Signaling Pathways by Selenomethionine

Mitochondria are important organelles for energy production within the cell and are the regulatory center for cell survival and death. Cytokines, death receptors, lipolytic hormones, pathogenic microorganisms, multiple metal ions, and radiation can affect the electron transport chain and energy metabolism within mitochondria, leading to apoptosis [23]. Studies have demonstrated that mitochondrial outer membrane permeabilization (MOMP) is the single most important link in most apoptotic signaling cascades. The mitochondria-mediated caspasease activation pathway is the major apoptotic pathway and is characterized by the permeabilization of the outer mitochondrial membrane and the subsequent release of cytochrome C into the cytoplasm to activate caspasease. The mitochondrial apoptotic pathway is coregulated by protein–protein interactions between members of the Bcl-2 protein family [35]. When cells are stimulated by multiple endogenous apoptotic signals (e.g., cytokine deficiency, drug induction, etc.), members of the Bcl-2 family of proteins regulate the irreversible over-opening of the mitochondrial permeability transition pore (MPTP). This leads to cytochrome C in the membrane interstitium (entering the cytosol to bind to apoptosis protease-activating factor-1 (Apaf-1), which recruits the Caspase-9 precursor, allowing it to be activated by self-shearing [36,37]. Activated Caspase-9 activates a downstream Caspase-3-mediated cascade reaction to cause apoptosis. In our experiments, adenovirus infection upregulated phosphorylated Caspase-9 to induce apoptosis, whereas selenomethionine treatment upregulated the expression of BCL-XL and BCL 2 and inhibited apoptosis.

**Figure 6:**
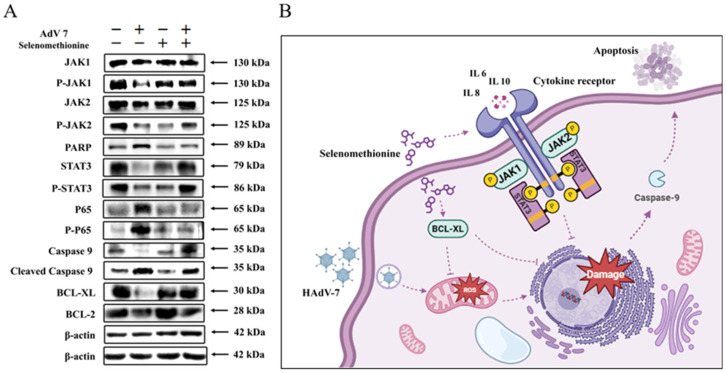
The inhibition of apoptosis signaling pathways by selenomethionine. (**A**) Selenomethionine inhibits HAdV-7-induced apoptosis through the JAK-STAT3 signaling pathway mediated by ROS. The concentrations of selenomethionine were 32 μM. (**B**) A diagram of the mechanism by which selenomethionine exhibits anti-HAdV-7 activity by modulating apoptotic signaling pathways.

Oxidative stress can induce inflammatory response, which can also aggravate oxidative stress. After viral infection, host cells will be stimulated to release a variety of pro-inflammatory cytokines, and defense cells will be induced to gather at the inflammatory site. Defense cells can resist bacterial invasion by producing a large number of reactive oxygen species, but at the same time, excessive reactive oxygen species will be produced, resulting in oxidative stress in tissues. IL-6 is a pleiotropic pro-inflammatory cytokine that not only affects the immune system but also plays a role in other systems and physiological mechanisms, such as the regulation of cell growth, as well as cellular activation, proliferation, survival, and differentiation. IL-6 stimulates the production of acute-phase proteins by a variety of leukocytes in the liver and is also particularly important in inducing B-cells to differentiate into antibody-forming cells (plasma cells). The binding of IL-6 to its receptor initiates intercellular communication, including JAK (Janus Kinase) kinase activation and Ras-mediated signaling activation. Pro-inflammatory cytokine levels are elevated after viral infection and decrease after viral clearance [32,38]. The Jak/Stat pathway acts as an inflammatory signaling pathway for stress; it regulates cell growth, differentiation, survival, and pathogen resistance. This pathway involves the IL-6 (gp130) family of receptors. When multiple cytokines/growth factors bind to the receptor, they can phosphorylate and activate JAK, which can phosphorylate tyrosine residues of downstream target proteins and recruit and phosphorylate the transcription factor STAT3. This allows STAT3 to enter the cell nucleus in the form of a dimer and bind to target genes, regulate the transcription of downstream genes, and control the processes of cell proliferation, differentiation, and apoptosis [39]. This experiment proved that selenomethionine can inhibit apoptosis by regulating the expression of Jak1/2 and STAT3 and then realize the inhibition of cell death. And IL-6 plays an important role in it. Our experimental results are consistent with the theoretical analysis (Figure 6A). 

Based on our results, we will further investigate the conversion of selenomethionine to selenocysteine to exert an indirect antiviral effect mechanism in the form of selenoproteins in future experiments.

## 3. Materials and Methods

### 3.1. Materials

The A549 cell line utilized in this investigation was acquired from ATCC, lyophilized in our lab using liquid nitrogen tanks, and cultivated in accordance with ATCC guidelines. A549 cells were cultured in serum-free minimum essential medium (MEM) (Gibco, Waltham, MA, USA) at 5% CO_2_, 37 °C. rAD7EGFP is a human adenovirus type 7 virus strain inserted with the *eGFP* gene that expresses green fluorescent eGFP during viral replication and proliferation. It was kindly donated by Prof. Zhou Rong’s group at the Institute of Respiratory Diseases, the First Affiliated Hospital of Guangzhou Medical University. The HAdV-7 virus strain was a clinical respiratory throat swab specimen provided by the Guangzhou Women and Children’s Medical Centre affiliated with Guangzhou Medical University. The experiments involved selenium-containing drugs, synthesized and benefited from the group of Prof. Chen Tianfeng, School of Chemistry and Materials, Jinan University.

### 3.2. Detection of Selenomethionine Toxicity by CCK-8 Assay

Cell Counting Kit-8 (CCK-8) (Beyotime Biotechnology, Haimen, China) is a WST-8-based assay for analyzing cell proliferation and cytotoxicity. The principle is that in the presence of electronically coupled reagents, a substance such as WST-8 can be reduced intracellularly by reductase to an orange-colored, dirty product. The number of metazoans produced is proportional to the number of living cells. Therefore, the toxicity of selenomethionine to A549 cells was determined by detecting the change in cellular absorbance, thus screening for a relatively safe range of drug concentrations [40].

A549 cells were spread in 96-well plates at a density of 7 × 10^4^/mL and incubated at 37 °C in a 5% CO_2_ incubator for 24 h until the cell density reached 80%. Selenomethionine with a concentration gradient ranging from 1 to 32 μM was prepared by diluting a 5 mM selenomethionine stock solution using DMEM (Thermo Fisher Scientific, Waltham, MA, USA). A total of 100 μL of selenomethionine dilution was added to each well and incubated at 37 °C, 5% CO_2_ for 48 h. CCK-8 working solution and DMEM were mixed in the ratio of 1:10, and 100 μL of CCK-8 dilution was added to each well. The incubation was continued for 30 min-2 h at 37 °C in a 5% CO_2_ incubator, and then the absorbance was detected at 450 nm using a fully automated enzyme labeling instrument.

### 3.3. TCID50 Determination of HAdV-7 and rAD7EGFP Viruses

For a period of 12 to 24 h, 8 × 10^4^ cells/mL of A549 cells was introduced to 96-well cell culture plates. The virus was diluted with pure DMEM in a 10-fold gradient to 10^−2^, 10^−3^, 10^−4^, 10^−5^, 10^−6^, 10^−7^, and 10^−8^, and 8 replicate wells were prepared for each gradient. A total of 100 μL of virus dilution was added to each well and incubated in a 37 °C CO_2_ incubator for 2 h to fully adsorb the virus onto the cells. The virus dilution was discarded, and 100 μL of 1% FBS + DMEM was added to each well to continue incubation in a 37 °C CO_2_ incubator.

### 3.4. Fluorescence Microscopy of Antiviral Efficiency 

In this experiment, rAD7EGFP, a HAdV-7 inserted with an *eGFP* gene, was applied. rAD7EGFP excites enhanced green fluorescence during in vivo replication, which enables the real-time dynamic observation of the process of the viral infection of the host cell, and the viral load is positively proportional to the fluorescence intensity [41].

Plates were spread at 7 × 10^4^ A549 cells/mL and incubated for 24 h until the cell density reached 80%. The virus was diluted with virus maintenance solution to 100 TCID_50_/0.1 mL, and then 100 μL of virus dilution was added to each well. The 96-well plates were cultured for 2 h under appropriate conditions, so that the virus was completely adsorbed on the cells. A549 cells were rinsed with PBS to eliminate any unadsorbed virus. Selenomethionine dilutions with a concentration gradient ranging from 1 to 8 μM were prepared by diluting a stock solution of 5mM selenomethionine using DMEM. After adding different concentrations of selenomethionine, the culture plates were incubated for 48 h under standard conditions. Finally, the fluorescence morphological changes in rAD7EGFP-infected A549 cells were observed by fluorescence microscopy (Leica, Wetzlar, Germany).

### 3.5. Detection of Cell Membrane Permeability and Phosphatidylserine Ectopy by Flow Cytometry

In normal cells, phosphatidylserine is found inside the cell membrane. In the early stage of apoptosis, the surface of the cell membrane is overturned, and the phosphatidylserine (PS) is flipped. Annexin V is a calcium-dependent phospholipid-binding protein that binds to exposed PS with high affinity. FITC-labelled Annexin V was used to detect early apoptotic cells in various cell types [42,43,44]. Cells stained with membrane-associated protein V can also be readily analyzed by fluorescence microscopy or flow cytometry. To assess the binding of membrane-associated protein V to apoptotic cells, the cells were cultured in six-well plates at a density of 8 × 10^4^ cells/mL under standard conditions. For in situ staining, 195 μL of Annexin V-FITC binding solution was added to each well, and 5 μL of Annexin V-FITC staining solution was added after mixing. Since PI is a red fluorescent dye, it cannot normally pass through cell membranes to stain the nucleus. Only necrotic cells or apoptotic cells undergoing secondary necrosis take up this dye. To identify cells undergoing necrosis or secondary necrosis, the material was thoroughly mixed before gently adding 10 μL of propidium iodide (PI) staining solution. The mixture was allowed to sit at room temperature for five to fifteen minutes. The Annexin-V/PI Co-Staining Kit can be used to determine the damage and apoptosis level of the cells, and then the results can be observed under the fluorescence microscope.

### 3.6. TUNEL-DAPI Double Staining Method

When apoptosis occurs, it activates the intracellular DNA endonuclease, which cuts the genomic DNA to produce a large number of sticky 3′-OH ends. The exposed 3′-OH terminus can be catalytically coupled with fluorescein, biotin-labeled dUTP by deoxyribonucleotide terminal transferase [44]. Thus, apoptosis can be detected by fluorescence microscopy or flow cytometry. To detect the status of DNA in the nucleus, DAPI was used to localize the nucleus. dUTP labeled with TUNEL-FITC can detect the location of the end of the nick of broken genomic DNA, indicating DNA damage in A549 cells [45].

After being cleaned, A549 cells were fixed for 30 min with 4% paraformaldehyde. A PBS solution containing 0.3% Triton X-100 was added once the fixative was removed, and then we let it sit at room temperature for five minutes. Then, 50 μL of TUNEL staining solution was added to each well and incubated for 1h. Subsequently, 10 μL of 10 μg/mL DAPI staining solution was added and incubated for 30 min after the addition of TUNEL staining solution for 30 min. The cells were washed with PBS to remove free TUNEL staining solution and DAPI staining solution, and the stained cells were kept on coverslips. The green and blue fluorescence changes in the cells were observed under a fluorescence microscope so as to determine the extent of DNA damage after the HAdV-7 infection of A549 cells.

### 3.7. Detection of Cytokines

In this experiment, cytokines were captured by AimPlex flow multifactor assay using artificial microspheres, and PE-labeled antibodies could bind to microsphere-bound cytokines [46,47]. The corresponding PE fluorescence intensity was converted to cytokine levels using flow cytometry for quantitative analysis.

Cell culture supernatants were collected from each group of HAdV-7-infected and selenomethionine-treated cells. Then, the cytokines in the cell culture supernatants were determined by microsphere capture assay and double antibody sandwich assay. The final experimental results were obtained by analyzing the data from the BD FACS Canto II flow cytometer by FCAP Array v3 software.

### 3.8. Detection of Mitochondrial Membrane Potential

Mitochondrial membrane potential is an important indicator of cellular health and functional status. JC-1 is an ideal fluorescent probe for detecting mitochondrial membrane potential (∆Ψm). It can detect changes in mitochondrial membrane potential within cells, so it is widely used to study mitochondrial behaviors related to cellular pathways, especially apoptosis. JC-1 is a dual fluorescent probe with two forms with similar structures but different fluorescence properties: the JC-1 aggregated (aggregate) and the JC-1 monomer (monomer) forms. Mitochondrial membrane potential is high in normal cells, and JC-1 exists in the matrix of mitochondria as a multimer that produces red fluorescence. In early apoptotic cells, the mitochondrial membrane potential is lowered, and JC-1 exists in the mitochondrial matrix in the form of monomers, producing green fluorescence. The change in JC-1 from red fluorescence to green fluorescence can reflect the decrease in cellular mitochondrial membrane potential. Therefore, the change in JC-1 fluorescence color can be used as a detection marker for the early stage of apoptosis [48,49].

After resuspension in 1% FBS + DMEM media, 500 μL of JC-1 staining working solution was added, and the mixture was sufficiently mixed before the cells were incubated for 20 min. At the end of the incubation, centrifuge and discard the supernatant, add 1 mL of JC-1 staining buffer and washed twice to remove the free JC-1 fluorescent probe. The staining results of the JC-1 monomer were detected in the FITC green fluorescence channel and JC-1 polymer in the PE red fluorescence channel using a BD FACS Canto II flow cytometer. Thus, the changes in the mitochondrial membrane potential of A549 cells were detected. The staining results can also be observed under the fluorescence microscope.

### 3.9. Detection of Reactive Oxygen Species (ROS) Levels

One often-used technique to measure intracellular reactive oxygen species levels is the Reactive Oxygen Assay. It is based on the fluorescent dye DCFH-DA’s variation in fluorescence intensity. Intracellular reactive oxygen species can oxidize non-fluorescent DCFH to produce fluorescent DCF. The amount of reactive oxygen species is correlated with the intensity of green fluorescence [50,51]. The detection of DCF fluorescence can detect the production of ROS in A549 cells.

After extracting 1 × 10^6^ A549 cells, 500 μL of DCFH-DA staining solution was added, and the mixture was incubated for 30 min. Cells were washed after centrifugation to remove DCFH-DA that did not enter the cells. The fluorescence intensity in A549 cells was monitored by a fully automated enzyme labeling instrument at an excitation wavelength of 488 nm and an emission wavelength of 525 nm. The staining results were observed by fluorescence microscopy.

### 3.10. Western Blot

The A549 cells infected with HAdV-7 were treated with selenomethionine for 48 h, and the protein concentration was determined by the BCA method. The protein sample was taken and added to double the volume of sample buffer and placed in a microtiter shock heater to denature at 95 °C for 5 min. The 10× Tris-Glycine-SDS (TGS) electrophoresis buffer was diluted to a 1× electrophoresis buffer containing 25 mM Tris, 192 mM glycine, and 0.1% SDS at pH approximately 8.6. The voltage was set, and electrophoresis started until the bromophenol blue reached the bottom of the separation gel. The membrane was transferred by electrophoresis under constant pressure at 110 V for 75 min. Successful transfer was indicated when a clear marker appeared on the PVDF membrane. The PVDF membrane was placed in a 5% milk containment solution and incubated for 1 h. The corresponding primary antibody was added and shaken at 4 °C overnight. The corresponding secondary antibody dilution was added and incubated at 4 °C for 2 h. The ECL chemiluminescence solution was prepared, and the results were observed with a chemiluminescence visualizer.

### 3.11. Statistical Analysis

The statistical analysis of data was performed using GraphPad Prism 8.0. The measurements are shown as the mean ± standard deviation for every group. A one-way ANOVA was utilized for comparisons between three or more groups, and independent *t*-tests or Mann–Whitney nonparametric tests were employed to evaluate the means between two sets of data. Significant statistical differences were observed between data when *p* < 0.05 (*), *p* < 0.01 (**), *p* < 0.001 (***), or *p* < 0.0001 (****).

## 4. Conclusions

In summary, in this study, selenomethionine possessed strong antioxidant properties and showed good antiviral ability in preventing adenovirus infection. The antiviral mechanism indicated that selenomethionine improved the regulation of ROS-mediated apoptosis by modulating the expression of Jak1/2, STAT3, and BCL-XL, which led to the inhibition of apoptosis. In conclusion, the present study illustrates that selenomethionine helps to control the overproduction of free radicals at sites of inflammation in the body, thereby protecting healthy cells from reactive oxygen species-induced oxidative damage. It may lead to new ways of controlling adenovirus infections, which is crucial for the development of new antiviral therapies.

## Data Availability

Data sharing does not apply to this article as no datasets were generated or analyzed during the current study.
