# Correction: Li et al. Selenomethionine Inhibited HADV-Induced Apoptosis Mediated by ROS through the JAK-STAT3 Signaling Pathway. *Nutrients* 2024, *16*, 1966

**DOI:** 10.3390/nu16172969

**Published:** 2024-09-03

**Authors:** Chuqing Li, Xia Liu, Jiali Li, Jia Lai, Jingyao Su, Bing Zhu, Buyun Gao, Yinghua Li, Mingqi Zhao

**Affiliations:** 1 Center Laboratory, Guangzhou Women and Children’s Medical Center, Guangzhou Medical University, Guangzhou 510120, China; lcq@stu.gzhmu.edu.cn (C.L.); liuxia@stu.gzhmu.edu.cn (X.L.); 2023210292@stu.gzhmu.edu.cn (J.L.); 2022210293@stu.gzhmu.edu.cn (J.L.); sujingyao@stu.gzhmu.edu.cn (J.S.); zhubing@gzhmu.edu.cn (B.Z.); 2 School of Pharmacy, Fudan University, Shanghai 200437, China; 19301030054@fudan.edu.cn

**Error in Figure**

In the original publication [1], there was a mistake in Figure 2A (the 32 µM group) as published. Due to the haste of proofreading, we accidentally mixed up these pictures. We have replaced the correct image of the 32 µM group. The corrected Figure 2A (the 32 µM group) appears below.


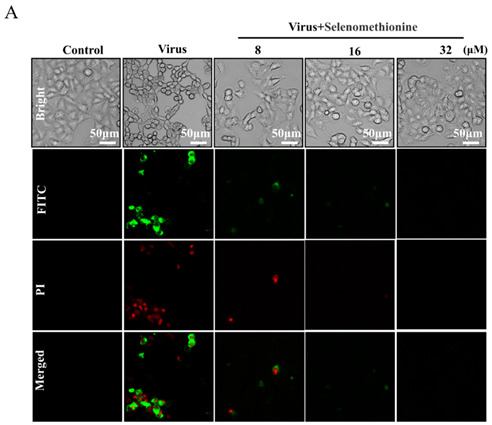


The authors state that the scientific conclusions are unaffected. This correction was approved by the Academic Editor. The original publication has also been updated.
